# A coalescent-based estimator of genetic drift, and acoustic divergence in the *Pteronotus parnellii* species complex

**DOI:** 10.1038/s41437-018-0129-3

**Published:** 2018-08-17

**Authors:** Liliana M. Dávalos, Winston C. Lancaster, Miguel S. Núñez-Novas, Yolanda M. León, Bonnie Lei, Jon Flanders, Amy L. Russell

**Affiliations:** 1 Department of Ecology and Evolution and Consortium for Inter-Disciplinary Environmental Research, Stony Brook University, Stony Brook, NY 11794 USA; 2 Department of Biological Sciences, California State University, Sacramento, CA 95819 USA; 3 Museo Nacional de Historia Natural Profesor Eugenio De Jesús Marcano. César Nicolás Penson Street esq. Máximo Gómez, Plaza de la Cultura, Santo Domingo, Dominican Republic; 4 Instituto Tecnológico de Santo Domingo y Grupo Jaragua, Santo Domingo, Dominican Republic; 5 Department of Organismic and Evolutionary Biology, Harvard University, Cambridge, MA 02138 USA; 6 School of Biological Sciences, Life Sciences Building, University of Bristol, 24 Tyndall Avenue, Bristol, BS8 1TQ UK; 7 Bat Conservation International, 500 North Capital of Texas Highway, Austin, TX 78746 USA; 8 Department of Biology, Grand Valley State University, Allendale, MI 49401 USA; 9 Present Address: Department of Biology, University of Alabama, Birmingham, AL 35294 USA; 10 Present Address: Microsoft, One Microsoft Way, Redmond, WA 98052 USA

**Keywords:** Population genetics, Evolutionary genetics

## Abstract

Determining the processes responsible for phenotypic variation is one of the central tasks of evolutionary biology. While the importance of acoustic traits for foraging and communication in echolocating mammals suggests adaptation, the seldom-tested null hypothesis to explain trait divergence is genetic drift. Here we derive *F*_ST_ values from multi-locus coalescent isolation-with-migration models, and couple them with estimates of quantitative trait divergence, or *P*_ST_, to test drift as the evolutionary process responsible for phenotypic divergence in island populations of the *Pteronotus parnellii* species complex. Compared to traditional comparisons of *P*_ST_ to *F*_ST_, the migration-based estimates of *F*_ST_ are unidirectional instead of bidirectional, simultaneously integrate variation among loci and individuals, and posterior densities of *P*_ST_ and *F*_ST_ can be compared directly. We found the evolution of higher call frequencies is inconsistent with genetic drift for the Hispaniolan population, despite many generations of isolation from its Puerto Rican counterpart. While the Hispaniolan population displays dimorphism in call frequencies, the higher frequency of the females is incompatible with sexual selection. Instead, cultural drift toward higher frequencies among Hispaniolan females might explain the divergence. By integrating Bayesian coalescent and trait analyses, this study demonstrates a powerful approach to testing genetic drift as the default evolutionary mechanism of trait differentiation between populations.

## Introduction

Determining the evolutionary processes shaping phenotypes has been a central task of genetics since its inception (Wright 1931). While traits such as body size or structure of acoustic calls have obvious implications for fitness (Brommer et al. 2014; Campbell et al. 2010; Kingston and Rossiter 2004; Puechmaille et al. 2014), genetic drift must first be tested as a potential primary evolutionary mechanism operating within and between populations. Echolocating mammals use acoustic calls for spatial orientation, foraging, and communication (Knörnschild et al. 2012), hence, acoustic traits should be subject to strong selection. Thus, the relative contributions of genetic isolation and resulting drift vs. adaptive, social, or sexual selection to acoustic traits becomes a central question in the evolutionary ecology of echolocating organisms.

In the absence of selection, traits—including acoustic traits—will evolve through neutral processes, reflecting population genetic structure and effective population size (Armstrong and Coles 2007; Chen et al. 2009; Odendaal et al. 2014). Very few studies of acoustic traits, however, directly evaluate predictions from drift models of trait evolution (e.g., Campbell et al. (2010)). Instead of modeling the amount of trait divergence attributable to neutral processes, given the evolutionary history of the lineage in question, qualitative assessments of genetic divergence compared to call frequency tend to be interpreted as reflecting some combination of neutral and selective processes (Clare et al. 2013). Additionally, constraints on acoustic traits arising from body size must be considered when analyzing the evolution of calls. Other things being equal, smaller bats will emit calls at higher frequencies than larger bats, in a pattern resulting from the acoustic properties of any physical object (Jones 1996). It is therefore important to include other correlated traits, such as body size, in analyses of mammalian echolocation traits.

Among bats, new phylogenies coupled with comparative analyses reveal ecological convergence in echolocation traits, including the evolution of highly sophisticated constant-frequency echolocation (Davies et al. 2012). Instead of emitting calls sweeping across multiple frequencies—or frequency modulated (FM) echolocation—, bats that use constant-frequency (CF) echolocation emit a pure tone at a constant frequency, ending with a FM sweep. Constant frequency calls are both simple and highly constrained (Kingston et al. 2001), and therefore easy to characterize by their dominant frequency.

In the New World, only the noctilionoid *Pteronotus parnellii* species complex has evolved constant frequency echolocation with Doppler-shift compensation (Clare et al. 2013). Recent research on the call frequencies of different populations in this species complex has focused on their correspondence with systematics (Clare et al. 2013; López-Baucells et al. 2017; Pavan et al. 2018; Pavan and Marroig 2016), but not on the evolutionary processes responsible for shaping these traits. Here we integrate population and quantitative genetic models to test drift as the primary driver of acoustic divergence in sister populations in the *P*. *parnellii* species complex. As island populations originating through over-water dispersal (Dávalos 2006; Pavan and Marroig 2017), the two focal populations have likely been subject to random genetic drift for at least part of their evolutionary history. We couple the isolation-with-migration model (Hey and Nielsen 2007) with quantitative analyses of phenotypic divergence codified by Spitze (1993) based on Lande’s (1992) interpretation of Wright’s (1951) island model, and extended to account for uncertainty in trait heritability and scaling by Leinonen et al. (2006). The methods presented here can be easily extended to test drift as an evolutionary mechanism across populations with known isolation-with-migration parameters (Muscarella et al. 2011; Russell et al. 2008).

## Methods

### Study sites, capture, and sampling methods

A total of 61 *Pteronotus pusillus* and *P. portoricensis* individuals were captured using a harp trap at the entrance of 6 caves distributed throughout the Dominican Republic on the island of Hispaniola (*P. pusillus*) and Puerto Rico (*P. portoricensis*, Supplementary Tables 1 and 2) (Núñez-Novas et al. 2016; Pavan and Marroig 2016). For ease in communication, we refer to individuals from both populations as *Pteronotus parnellii* species complex or *sensu lato* (s.l.). Within minutes of capture, skin tissue was sampled using an Acu-Punch sterile, disposable 2-mm skin biopsy punch (Acuderm, Inc.). Punctures were transferred to ~0.7 g indicator silica for desiccation and transport (Corthals et al. 2015). Bats were released at the site of capture within 60 min of being caught.

### Morphological and echolocation data collection

After sexing, standard mammalian measurements were collected comprising length of body, tail, ear, foot, and forearm, and body mass. We recorded biosonar vocalizations from bats in a wire mesh cage (approximately 15 × 15 × 35 cm). Recordings were made with a Larson Davis 6.25 mm instrumentation microphone (model no. 2520) and Larson Davis preamplifier (model no. PRM 422), and amplified with a PCB Piezotronics Signal Conditioner (model no. 480E09). Calls were recorded onto a laptop computer using a National Instruments A–D card (DAQCard-6062E), recording at 356 kHz using BatSound Pro v3.31 (Pettersson Elektronik). We placed bats into the cage and allowed them to calm briefly. With the microphone held ~10 cm from the bat’s head, we recorded three to five files of 5 s in duration.

We analysed the vocalizations using the callViewer-18 script compiled with Matlab by Mark Skowronski. The spectrogram parameters of the program were set at a window length of 3 ms and an FFT size of 16,384 points resulting in a resolution of 22 Hz. Automated detection parameters set a frame rate of 10,000 frames per second (resulting in a frame duration of 0.1 ms), a bandpass filter between 20 and 130 kHz, and a minimum energy of 45 dB. We focused frequency analysis on the constant frequency (CF) portion of the second harmonic of the calls, as this is known to be the highest amplitude and most consistent component of the biosonar calls of this species, and relates directly to the best frequency of the cochlea, auditory nerve, and central nervous system (Huffman and Henson 1990).

Calls were selected for analysis based on the best available signal-to-noise ratio. Approximately 65 individual calls were identified for each bat and targeted for frequency analysis in callViewer-18. The resulting tables assigned a number to each call, the time to the centre of each analysis frame, and the frequency of that frame and its slope (kHz/ms), among other parameters. Segments of calls were included in analysis when their slopes were zero for a minimum of 1 ms (10 consecutive frames). Within a single call, variations in the frequency of the CF component were accepted if they shifted within one increment of resolution, provided that the shift persisted for a minimum of 1 ms. These variations correspond to Doppler-shifts induced by motion of the bat in the cage. All frames of a call deemed to be of constant frequency were averaged and this value was taken as the frequency of the CF portion of the call.

### Molecular data collection

DNA was extracted from the desiccated skin samples using QIAmp or DNeasy extraction kits (Qiagen, Inc.), and following the manufacturer’s protocol for animal tissues (Corthals et al. 2015). Extracted DNA was used as a template in PCR amplifications using *Taq* polymerase and primers for the entire mitochondrial *cytochrome b* (*cytb*) gene, and partial sequences from four nuclear loci: *stat5a, plcb4, rag2*, and *atp7a*. Primers and amplification conditions for each locus have been described in detail elsewhere (Dávalos et al. 2014).

### Population genetic analyses

Aligned datasets for each locus were edited by eye to remove sites violating the infinite sites model (i.e., sites with >2 character states). The remaining data were then filtered using the Perl script IMgc (Woerner et al. 2007) to yield the longest non-recombining fragment for each locus. The filtered data included 1,121 bp for *cytb* (*n*_DR_ = 26, *n*_PR_ = 6), 472 bp for *rag2* (*n*_DR_ = 34, *n*_PR_ = 28), 307 bp for *plcb4* (*n*_DR_ = 26, *n*_PR_ = 24), 429 bp for *stat5a* (*n*_DR_ = 10, *n*_PR_ = 12), and 637 bp for *atp7a* (*n*_DR_ = 2, *n*_PR_ = 8). Sample sizes for the nuclear loci are given as numbers of sequenced chromosomes. McDonald and Kreitman (1991) tests were conducted on all coding regions (*cytb, atp7a*, and *rag2*); in no case did we find evidence of selection (Supplementary Table 3).

Previous analyses indicated the two populations are completely discontinuous (Pavan and Marroig 2016), and our own analyses failed to show consistent differentiation among sampling sites within islands. The historical demography of these populations was therefore estimated using the two-population model of IMa2 v.8.27.12 (Hey and Nielsen 2004). As implemented here, this model estimated six parameters: *θ* (=4*N*_e_μ) for each of the populations from Hispaniola, Puerto Rico, and the most recent common ancestor, *m* (=*M*_i_/μ, where *M*_i_ is the rate of migration into population i) in each direction, and τ (=*t*μ, where *t* is the time since population splitting). Priors were set to uniform distributions (U) (0,50) for *θ* and migration parameters, and U(0,5) for τ. Five independent runs were allowed to burn-in for ~5 million steps each, after which each Markov chain Monte Carlo (MCMC) search was allowed to continue for ~10 million steps. Each run consisted of 30 heated chains with heating parameters *h*_a_ = 0.975 and *h*_b_ = 0.75. Relative substitution rates for each locus were estimated in IMa2 and converted to substitutions/site/year based on a *cytb* rate of 2.99 × 10^−8^ substitutions/site/year from a fossil-calibrated phylogenetic analysis of Noctilionoidea including *P. parnellii* s.l. (Rojas et al. 2016). Coalescent-scaled parameters (*θ*, *m*, and τ) were converted to natural parameters (*N*_e_, *M*_i_, and *t*, in order) as described above using the geometric mean of locus-specific substitution rates (=5.67 × 10^−7^ substitutions/locus/year) and a generation time of 2 years. After verifying that all runs converged on similar posterior distributions, we used IMa2 to calculate the joint posterior densities for each parameter based on the 47,983 coalescent genealogies resulting from all five separate runs.

We estimated fixation index, or *F*_*ST*_ values (Wright 1965), based on the posterior densities of directional effective number of migrants (*N*_*e*_*m* or *Nm*) from IMa2 (Supplementary Figure 1). The transformation to obtain *F*_*ST*_ values was based on Wright’s (1965) island model with Takahata’s (1983) correction for a finite number (*d*) of populations, in which:

1 $$F_{{\rm{ST}}} = \frac{1}{{1 + 4N_{\rm{e}}m\left( {\frac{{d^2}}{{\left( {d - 1} \right)^2}}} \right)}}.$$

While attempts to estimate *Nm* from *F*_*ST*_ values have long been criticized as overly simplistic (Whitlock and McCauley 1999), here we instead use this relationship to transform a posterior distribution of *Nm* into a posterior distribution of *F*_ST_ values. This transformation accounts for the variance in *F*_*ST*_ arising from stochastic errors as well as differences between loci, which are overlooked when transforming *F*_ST_ into migration rates. In addition, by estimating directional values of *Nm* using a non-equilibrium coalescent-based method, we avoid the assumptions of equal *N*_e_ and *m* for all populations, as well as the condition of mutation-drift-migration equilibrium for the entire system.

In addition to obtaining *F*_ST_ distributions from coalescent posterior distributions of *Nm*, we estimated global *G′*_ST_ (equivalent to *F*_ST_) from the multi-locus sequence data, including a correction for finite number of subpopulations (Hedrick 2005), and then bootstrapped to obtain a distribution around that value using the R package *mmod* v. 1.3.3 (Winter 2012). This provided a direct comparison between our coalescent-based method and previous methods of characterizing neutral variation within the genome.

### Quantitative trait comparisons

All statistical analyses were conducted in the R statistical language v.3.4.4. We estimated the summary statistics of Hispaniolan and Puerto Rican populations for call frequency, body mass, and forearm length phenotypes. Bayesian estimates of differences between populations were obtained using the *BEST* R package (Kruschke 2013).

### Models of echolocation frequency as a function of morphological data

The frequency of the CF portion of the second harmonic of a set of biosonar calls was analyzed for each bat, as described above. The median of these values was used to represent its CF in frequentist statistical analyses. Principal component analysis of morphological measurements was used to obtain orthogonal variables summarizing the variation in body size among individuals. We used linear models of CF call frequency as a function of principal components or body measurements to test whether variation in call frequency was explained by body size (Jones 1996).

### Using Bayesian models to estimate P_ST_ and compare to F_ST_

The divergence in a quantitative, heritable trait is expressed as an analogue of the *F*_*ST*_ called the *Q*_*ST*_, and given by:

2 $$Q_{{\rm{ST}}} = \frac{{\sigma _{A_{\rm{B}}}^2}}{{\sigma _{A_{\rm{B}}}^2 + 2\sigma _{A_{\rm{W}}}^2}};$$

in which $\sigma _{A_{\rm{B}}}^2$ is the additive variance for the trait between populations, and $\sigma _{A_{\rm{W}}}^2$ is the additive variance within populations. Genetic drift as a mechanism behind the quantitative differentiation is rejected when *Q*_ST_ exceeds *F*_ST_ from neutral loci. For most traits in wild populations, however, the additive genetic variance is unknown (Brommer 2011). In this case, the variance terms need to be scaled by a constant *c* and the heritability *h*^*2*^, resulting in the estimate of phenotypic differentiation, or *P*_*ST*_:

3 $$P_{ST} = \frac{{c\sigma _B^2}}{{c\sigma _B^2 + 2h^2\sigma _W^2}} = \frac{{\frac{c}{{h^2}}\sigma _B^2}}{{\frac{c}{{h^2}}\sigma _B^2 + 2\sigma _W^2}};$$

in which *c*/*h*^*2*^ is the additive genetic contribution to the proportion of the between-population variance. In most empirical cases the *c*/*h*^*2*^ ratio is unknown, but it determines how robust the *P*_*ST*_ approximation to the *Q*_*ST*_ is. If the *P*_*ST*_ exceeds the neutral expectation —the *F*_ST_—at *c* = *h*^*2*^, then it will also exceed this expectation when *c* > *h*^*2*^. However, when *c* < *h*^*2*^, there is a limit to the extent to which the *P*_ST_ reflects the *Q*_*ST*_ exceeding the neutral expectation. This critical value is estimated by calculating *c* / *h*^*2*^_critical_ (Eq. 13) for the lower 5% tail of *P*_ST_ and the upper 5% tail of *F*_*ST*_ distributions (Brommer 2011):

4 $$\frac{c}{{h^2}}_{{\rm{critical}}} = \frac{{2\sigma _{W0.05}^2F_{{\rm{ST}}\,0.95}}}{{\sigma _{B\,0.05}^2(1 - F_{{\rm{ST}}\,0.95})}} = \frac{{(1 - P_{{\rm{ST}}\,0.05})F_{{\rm{ST}}\,0.95}}}{{P_{{\rm{ST}}\,0.05}(1 - F_{{\rm{ST}}\,0.95})}}.$$

When *c* / *h*^*2*^_critical_ is low, there is a large range of *c* / *h*^*2*^ over which the phenotypic divergence (*Q*_ST_) will exceed the neutral genetic divergence (*F*_ST_), indicating the comparison is robust, and thus greater confidence in the inference of quantitative divergence exceeding neutral genetic divergence.

One advantage of our method emerges from obtaining *F*_*ST*_ values from posteriors for directional migration rates (Supplementary Figure 1). Unlike *F*_ST_ distributions based on assumptions of normality, the distribution of multi-locus *F*_ST_ values can be directly compared to the distribution of trait-specific *P*_ST_ values without assuming a canonical frequency distribution for either. Thus, we can also summarize the entire parameter-space over which any differences between *P*_ST_ and *F*_ST_ can be observed in the best-case scenario of *c* *=* *h*^*2*^. We use the frequency distributions of *F*_ST_ and trait-specific *P*_ST_ to visualize differences and test genetic drift as the evolutionary mechanism underlying differentiation in the two populations. The overlap between distributions was calculated by computing kernel densities on the same scale and their overlap using the R package *sfsmisc* v. 1.1-0 (Maechler 2016).

To calculate trait-specific *P*_ST_ values, we estimated the phenotypic variance between and within populations using linear hierarchical (or multilevel) models (Brommer et al. 2014). Multilevel models have the advantage of fitting parameters specific to clusters of observations (Gelman and Hill 2007), such as islands, without discarding the variation in observations of individuals. These models had the quantitative trait as a response variable, with population as an island-specific (or random) effect, and sex as either a sample-wide (or fixed) or an island-specific effect (including a potential interaction between sex and island). Including sex as an effect accounts for phenotypic variance between sexes that might otherwise obscure the pattern of variation between populations. The *P*_ST_ was estimated as a derived quantity by coding eq. 13 for *c* *=* *h*^*2*^, including the population-specific phenotypic variance $\sigma _B^2$, and the residual variance $\sigma _W^2$, after factoring out the effects of sex on the traits. In general, Bayesian analyses of the *Q*_ST_, approximated here by the *P*_ST_, increase precision in estimates of the entire posterior distribution (O’Hara and Merilä 2005).

Hierarchical models were coded in Jags v.3.3.0 (Plummer 2003), and ran in the R package *R2jags* v.0.04-01 (Su and Yajima 2012), with a burn-in of 25,000 iterations followed by 25,000 additional iterations. Posteriors were sampled every 25 generations to produce effective sampling sizes for the posterior of at least 1600, and assessed using the potential scale reduction factor (PSRF), which approaches 1 at convergence (Gelman and Rubin 1992). All posterior parameter estimates had PSRF ≤ 1.003. The prior for the population-specific effect was drawn from a normal distribution, with identical independent priors for between- and within-population variances set as half-Cauchy distributions with variance of at least 10,000. These priors are robust and do not make any assumptions about the relative contribution of variation from different levels in the hierarchy (Gelman and Hill 2007). In addition, we used estimates from these models to build posterior predictive checks, and compared the simulated data to parameters from the observations using the *bayesplot* R package (Gabry 2017). Estimates of *P*_*ST*_ with their posterior distribution are shown for the best-case scenario of *c* = *h*^*2*^.

## Results

### Population genetic analyses of isolation with migration

**Fig. 1:**
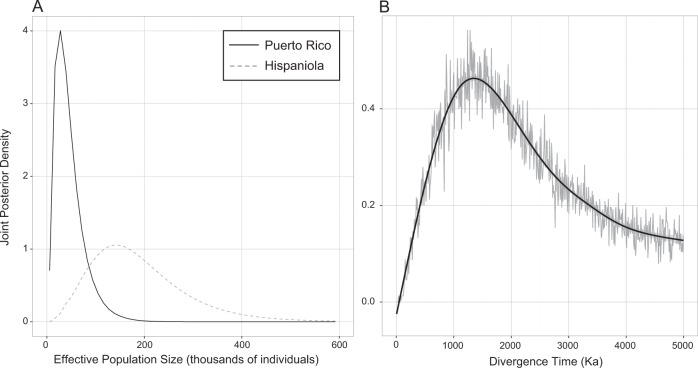
Results from IMa2 analyses of *Pteronotus parnellii* s.l. populations. **a** Joint posterior density of *N*_e_ estimates for island populations. **b** Divergence time estimates between Puerto Rican and Hispaniolan populations in thousands of years (Ka)

We estimated the historical demography of Hispaniolan and Puerto Rican populations of *Pteronotus parnellii* s.l. using the two-population model of IMa2. Five independent runs of ~10 million steps each converged on similar point estimates and posterior densities for all parameters except θ_MRCA_ (Supplementary Table 4). We focus here on the parameters of interest for the phenotypic evolution models: θ for each island population, directional migration rates (*m*_*i*_), and the splitting time (τ) for the two daughter populations. The scaled population size parameter for Hispaniola (θ_DR_ = 0.625, 95% high probability density [HPD] = 0.225, 1.975) was consistently estimated to be greater than that for Puerto Rico (*θ*_PR_ = 0.125, 95% HPD = 0.025, 0.575). Assuming a mean multilocus substitution rate of 1.13 × 10^−6^ substitutions/locus/generation, these θ estimates correspond to modal effective population sizes of ~ 138,000 individuals in the Hispaniolan population (95% HPD = 50k, 435k) and ~28,000 individuals in the Puerto Rican population (95% HPD = 6k, 127k; Fig. 1a). These results are consistent with our best knowledge of the biology of these species, namely, that members of the island *P. parnellii* species complex are of least conservation concern in their respective ranges (Schipper et al. 2008), numerous caves throughout their range are known to harbour thousands to tens of thousands of individuals (Gannon et al. 2005; Núñez-Novas et al. 2016), and the range of *P. pusillus* (Hispaniola) is much larger in area than the range of *P. portoricensis* (Puerto Rico). These analyses converged on an estimate of the scaled splitting time parameter τ = 0.703 (95% HPD = 0.258, 4.793); this corresponds to a splitting time of ~1.24 Ma (95% HPD = 0.45 Ma, 8.45 Ma; Fig. 1b). This estimated splitting time corresponds with recent, independently derived phylogenetic estimates of divergence of ~1.2 Ma between *P. portoricensis* and *P. pusillus* by Pavan and Marroig (2017). Migration rates were estimated as *m*_DR_ (the coalescent-scaled rate of migration from Puerto Rico into Hispaniola) = 1.875 (95% HPD = 0.475, 15.07) and *m*_PR_ = 0.025 (95% HPD = 0.025, 30.52). These estimates correspond to estimates of the effective number of migrants per generation (*Nm*) of *Nm*_DR_ = 0.325 (95% HPD = 0.077, 2.336) and *Nm*_PR_ = 0.049 (0.007, 0.907; Supplementary Figure 1).

### Relationship between echolocation frequency and body size

**Fig. 2:**
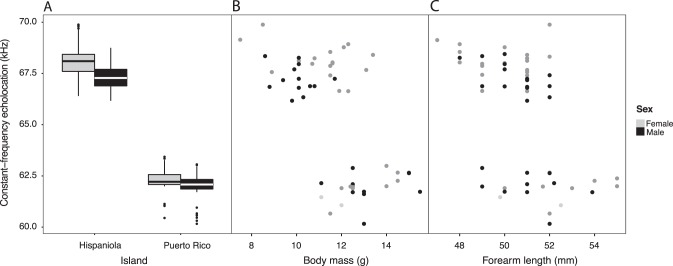
Echolocation call frequency by island by sex and its relation to body dimensions. **a** Boxplots of echolocation frequency (summarizing 10 calls/individual). Bayesian 95% high-probability density (HPD) of the difference in call frequency means between Puerto Rico and Hispaniola was 5.2–6.0 kHz. **b** Call frequency as a function of body mass. Analyses of covariance support very different call frequency for island groups (*F*_(1, 49)_ = 704.260, *P* value = 0.000), but no influence of body mass on the call frequency (*F*_(1, 49)_ = 0.435, *P* value = 0.512). The 95% HPD of population differences in means for body mass was 0.89–2.29 g. **c** Call frequency as a function of forearm length. Analyses of covariance support little influence of forearm length on the call frequency (*F*_(1, 52)_ = 2.851, *P* value = 0.097). The 95% HPD of population differences in means for forearm lengths was −1.82, 0.422 mm

Empirical frequency distributions of echolocation frequencies showed complete separation between Puerto Rican and Hispaniolan recordings (Fig. 2a). Analyses of covariance (ancova) revealed that call frequencies of Hispaniolan and Puerto Rican bats were significantly different (*F*_(1,49)_ ≥ 405.6, *P* value < 2e-16, Fig. 2a), but this variation was not a linear product of body size as measured by body mass (*F*_(1,49)_ = 0.436, *P* value = 0.512, Fig. 2b), forearm length (*F*_(1,52)_ = 2.01, *P* value = 0.162, Fig. 2c), or the first principal component of all external body measurements (*F*_(1,44)_ = 0.258, *P* value = 0.614, Supplementary Figure 2). The only variable to show a correlation with call frequencies was principal component 2, but it still did not explain the divergence between the two populations (PC2, *F*_(1,44)_ = 7.39, *P* value = 0.010, Supplementary Figure 2). The distributions of external body measurements for Hispaniolan and Puerto Rican populations overlapped almost completely (Fig. 2b). Using a Bayesian implementation of the *t* test (Kruschke 2013), we found Hispaniolan bats had a mean forearm length 0.71 mm shorter (HPD = −1.82, 0.422), confirming those bats are generally smaller but not significantly so (Fig. 2b). The first three principal components of morphological variation were able to discriminate between the Hispaniolan and Puerto Rican populations (manova of principal components of morphological measurements *F*_(1,46)_ = 17.46, *P* = 1.042e-07; Wilk’s Λ = 0.467, partial η^2^ = 0.53).

### Estimates of genotypic and phenotypic differentiation

**Fig. 3:**
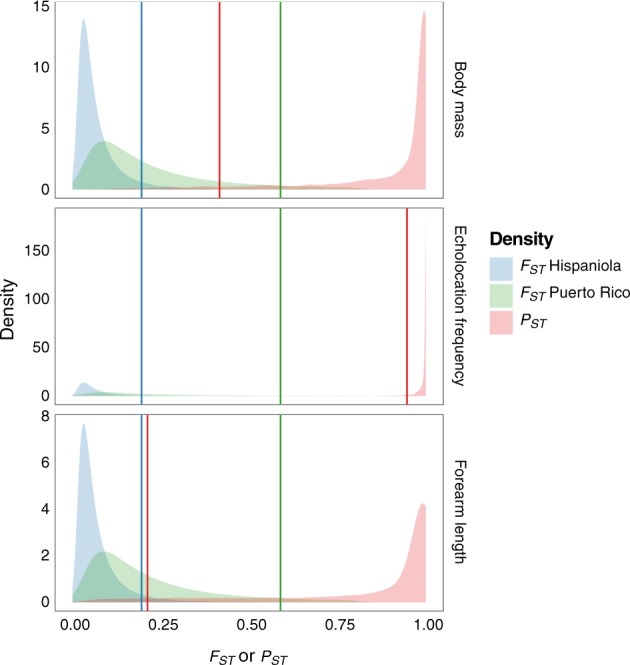
Densities of Bayesian posteriors for *F*_ST_ based on between-population migration rates, and *P*_ST_ for relevant phenotypic variables (Brommer et al. 2014). The lines show the 95th percentile for the corresponding *F*_*ST*_, and the 5% percentile for the *P*_ST_. The overlap between *P*_ST_ body mass and *F*_ST_ Hispaniola was 0.023, for *F*_ST_ Puerto Rico it was 0.084; between *P*_ST_ call frequency and *F*_ST_ Hispaniola was < 0.001, for *F*_ST_ Puerto Rico it was 0.003; and between *P*_ST_ forearm length and *F*_*ST*_ Hispaniola was 0.049, for *F*_ST_ Puerto Rico it was 0.125

The posterior distributions of population-specific migration rates were used to calculate *F*_*ST*_ values (Table 1, Fig. 3). While all Bayesian models for quantitative traits performed well in posterior predictive checks, modelling the island-specific effects of sex improved estimates of the variance for sexes on different islands (Supplementary Figure 3). Body size variables showed no sex-specific effects: the posterior distributions of the coefficients of the effect of being male had slightly negative means for each trait, but included zero (Table 1). Compared to females, Hispaniolan males called at lower frequency (Table 1, Supplementary Table 5, Fig. 2a). This effect persisted even after including body mass or forearm length as covariates of call frequency (Table 2).

**Table 1:** Posterior estimates of *F*_*ST*_ into each island, *G’*_ST_ (normalized multi-locus *F*_ST_), trait-specific *P*_ST_ with sample-wide effects of sex for each trait, and island-specific effects of sex in the case of call frequency

Variable	His. *c*/*h*^*2*^_*critical*_	P.R. *c*/*h*^*2*^_*critical*_	Mean	2.5% HPD	Median	97.5% HPD
Multi-locus *F*_*ST*_ Hispaniola	—	—	0.072	0.013	0.051	0.259
Multi-locus *F*_*ST*_ Puerto Rico	—	—	0.215	0.033	0.162	0.684
Multi-locus bidirectional *G’*_*ST*_ (confidence interval)	—		0.215	0.116	0.215	0.314
Body mass *P*_*ST*_	0.342	>1	0.893	0.302	0.987	1.000
Body mass sex effect	—	—	−0.621	−1.290	−0.618	0.038
Call frequency *P*_*ST*_	0.014	0.080	0.990	0.915	0.999	1.000
Call frequency sex effect Hispaniola	—	—	−0.736	−1.211	−0.736	−0.263
Call frequency sex effect Puerto Rico	—	—	−0.138	−0.752	−0.142	0.495
Correlation of variance between sex and island	—	—	−0.043	−0.981	−0.053	0.964
Forearm *P*_*ST*_	0.901	>1	0.835	0.135	0.979	1.000
Forearm sex effect	—	—	−0.220	−1.002	−0.218	0.550

The sex effect is coded with females as the baseline, the effect shown is for males. *c* / *h*^*2*^_critical_, critical value of the proportion of heritability ascribable to the additive genetic variance for the *P*_*ST*_ vs. *F*_*ST*_ comparison

*His.* Hispaniola, *HPD* high probability density, *P.R.* Puerto Rico

**Table 2:** Summary of posterior estimates of echolocation call frequency *P*_*ST*_ for analyses correcting for body size through island-specific effects of sex, and sample-wide effect of body mass (BM), or forearm length (FA)

Model	Variable	Mean	2.5% HPD	Median	97.5% HPD
Both	*P*_*ST*_	0.989	0.907	0.999	1.000
BM	Sex effect Hispaniola	−0.777	−1.279	−0.781	−0.249
BM	Sex effect Puerto Rico	−0.127	−0.736	−0.130	0.509
BM	Coefficient on body mass	−0.034	−0.190	−0.034	0.125
BM	Correlation of variance between sex and island	−0.050	−0.982	−0.083	0.961
FA	Sex effect Hispaniola	−0.685	−1.151	−0.682	−0.228
FA	Sex effect Puerto Rico	−0.285	−0.879	−0.297	−0.351
FA	Coefficient on forearm length	−0.102	−0.238	−0.102	0.034
FA	Correlation of variance between sex and island	−0.051	−0.980	−0.030	0.968

The sex effect is coded with females as the baseline, i.e., the effect shown is for males

*HPD* high probability density

Estimates of trait-specific *P*_*ST*_ at *c* = *h*^*2*^ showed the greatest differentiation in echolocation call frequency, with lower estimates for body mass and forearm length (Table 1). The call frequency *P*_*ST*_ for Hispaniola also had low *c*/*h*^*2*^_*critical*_ (0.014 with island-specific sex effect, 0.022 with sample-wide sex effect), while the *c* / *h*^*2*^_*critical*_ values of almost all other comparisons were several times larger, and even >1 for the body size data from Puerto Rico. Comparisons of the distributions of *P*_ST_ and *F*_*ST*_ show overlap in distributions ≥4.9% for body size variables and Puerto Rico, while the lowest overlap corresponded to *P*_ST_ call frequency and *F*_ST_ Hispaniola (0.02% with island-specific sex effect, Fig. 3, and 0.06% with sample-wide sex effect, Supplementary Figure 4). This indicates phenotypic differentiation was significantly greater than neutral genetic differentiation for call frequency trait in the Hispaniolan population.

## Discussion

We tested genetic drift as the evolutionary process underlying acoustic differentiation by integrating phenotypic and genotypic models, rejecting this evolutionary mechanism as a probable explanation for the divergent call in the Hispaniolan population. To this end, we integrated coalescent-based models and well-established methods for estimating *F*_*ST*_ (Wright 1965) to directly compare measures of differentiation. This approach accounts for variation in *F*_ST_ estimates in comparisons with *P*_ST_, allows the estimation of different *F*_ST_ distributions corresponding to directional rates of migration, and enables measuring overlap to quantify the correspondence between genotypic and phenotypic divergence. In addition, compared to a bootstrapped distribution around a point estimate of global *F*_ST_, the distributions of *F*_ST_ derived from Bayesian posterior estimates of *Nm* more realistically reflect the information content in the data.

### Comparisons of *F*_ST_ and *P*_ST_

Our comparisons differ from traditional comparisons of genotypic and phenotypic differentiation in two ways. First, calculating *F*_ST_ values from estimates of the number of migrants (*Nm*) overcomes the limitations of traditional approximations by integrating both stochastic variation from individual sampling and variance across loci and by allowing for asymmetric rates of gene flow (Muir et al. 2012; Sundqvist et al. 2016). Importantly, because *Nm* is a compound parameter ($4Nm = 4N_{\rm{e}}\mu \ast \frac{M}{\mu }$), these estimates are independent from the highly variable mutation rate. This approach, then, can test the genetic drift hypothesis for each population, with potential applications for testing the evolutionary processes behind differentiation in continuous traits across many populations in the West Indies and beyond (Muscarella et al. 2011; Russell et al. 2008). Second, *F*_ST_ values were derived from a Bayesian posterior of *Nm*, and the resulting distribution of *F*_ST_ was then compared against the posterior of phenotypic differentiation (Fig. 3). Therefore, the frequency distribution of *F*_ST_ does not need to be generated by bootstrapping, or some other means of introducing variation around a single point estimate. The difference between the frequency distributions of *F*_*ST*_ and *P*_*ST*_ can then be calculated as a derived quantity in Bayesian analyses or, in the case of overlap, as an estimate of the overlapping portion of the frequency distributions (Fig. 3). When estimates of neutral genetic and trait differentiation clearly overlap, as for body size variables for Puerto Rico (Fig. 3), the *c* / *h*^*2*^_critical_ value becomes irrelevant because the overlap between trait-specific *P*_*ST*_ values and estimates of genetic differentiation occurs under the best-case scenario of *c* *≥* *h*^*2*^. In cases in which the frequency distributions differ, as for call frequency and perhaps body mass (Fig. 3), the *c* / *h*^*2*^_critical_ value can be estimated with greater confidence on the upper tail of the *F*_ST_ than has been feasible before.

An analysis using the traditional method of estimating a distribution of genetic differentiation illustrates some benefits of the proposed approach. We estimated global *G*′_ST_ (equivalent to *F*_ST_) from the sequence data (Hedrick 2005), and bootstrapped to obtain a distribution around that value. With a mean *G’*_*ST*_ = 0.215 (normalized 95% CI: 0.116-0.314), and like the transformed Bayesian posterior distribution of *Nm* for Hispaniola, the global *G*′_ST_ shows no overlap with *P*_ST_ for the call frequency, but does overlap with *P*_ST_ for both body mass and forearm length. Unlike our approach using transformed posterior distributions, the traditional approach assumes an equilibrium model with gene flow being equal in both directions, and so would lead to rejection of genetic drift for the Puerto Rican population with a *c* / *h*^*2*^_critical_ value of 0.03. In contrast, the method presented here accounts for directional differences in *F*_*ST*_ that lead to a sixfold higher *c* / *h*^*2*^_critical_ for the Puerto Rican (0.080) compared to the Hispaniolan population (0.014), resulting in much greater robustness for the rejection of drift for the latter.

Our method of deriving *F*_ST_ distributions from Bayesian posterior distributions of *Nm* may be practically implemented in future studies. Here, we chose the IMa2 software package to estimate distributions of *Nm* because the underlying demographic model is directly applicable to the sampled populations of *P. portoricensis* and *P. pusillus*, two sister species with no genetic structure within islands and DNA sequence data well described by the infinite sites model. In other applications, more complex demographic scenarios and/or different data types may apply different methods to estimate the *Nm* posterior (e.g., approximate Bayesian methods). Regardless of the specific method used to estimate these posterior distributions, we urge that the data be tested for neutrality, that appropriate demographic models be used, and that appropriate substitution models be specified in the analysis. In addition, we expect that, regardless of the method used, Bayesian estimates of the *Nm* composite parameter will yield more precise posteriors with larger datasets, particularly with an increase in the number of variable markers rather than an increase in the number of sampled individuals (Knowles and Carstens 2007; Leaché et al. 2013).

### Acoustic divergence despite similar body size

Previous analyses had shown genetic differentiation between the Hispaniolan (*Pteronotus pusillus*) and Puerto Rican (*P. portoricensis*) populations in the *P. parnellii* species complex (Dávalos 2006; Pavan and Marroig 2016). We confirmed two independent, allopatric populations characterized by divergent acoustic signals. Acoustic differentiation cannot be explained by the subtle differences in body size that characterize the two populations. Linear combinations of morphological measurements of the skull or body can discriminate between species (Pavan and Marroig 2016), but if the two populations were sympatric they could not be easily distinguished using external measurements (Fig. 2b), and would be considered cryptic (Kingston et al. 2001). In contrast, differences in call frequency were large and consistently explained by population membership, but not by size (Fig. 2, Supplementary Figure 2). These observations raise the question of how the two sister populations evolved divergent call frequencies.

*Pteronotus pusillus* and *P. portoricensis* are allopatric, insular populations with a long history of isolation for approximately 1.2 million years (Fig. 1b). This long isolation coupled with little subsequent migration makes genetic drift an obvious mechanism for acoustic divergence (Supplementary Figure 1). Multiple adaptive, social, and sex-driven evolutionary causes have been invoked to explain variation in call frequency between populations and between species, including habitat physical features (Odendaal et al. 2014), ambient humidity (Guillén et al. 2000), acoustic environment (Gillam and McCracken 2007), ecological segregation (Kingston et al. 2001; Kingston and Rossiter 2004), female choice (Puechmaille et al. 2014), and cultural drift (Yoshino et al. 2008). Crucially, the genetic drift model is seldom tested in a way that considers more than simple pairwise genetic distances (e.g., Odendaal et al. 2014; Puechmaille et al. 2011; Yoshino et al. 2008). Here, the genetic drift hypothesis is rejected for Hispaniola over ~98% of the range of *c* / *h*^*2*^ < 1or additive proportion of heritability (Table 1, Fig. 3). This finding, along with sexual dimorphism in the Hispaniolan but not Puerto Rican population, detected even after accounting for body size, suggests sexual or social mechanisms could explain trait differentiation in these island populations.

As high-frequency calls are an honest signal of body condition, females seem to select for higher-frequency males in at least one constant-frequency echolocating bat species (Puechmaille et al. 2014). If that were the case here, female choice would lead to the higher frequency of *Pteronotus pusillus*. Male calls, however, were significantly *lower* in this population even after accounting for their somewhat smaller body size (Table 2). If this dimorphism is the result of female choice, then it runs counter to the direction of divergence that needs to be explained. The alternative is for females to drive the change in call frequency, not through sexual selection, but through cultural drift (Yoshino et al. 2008). In this case, the maternal transmission of culturally distinct higher-frequency calls coupled with female philopatry leads to long-term divergence in acoustic calls even after males disperse. Over the time of estimated isolation even subtle cultural differences together with overwater barriers could explain the divergence found. Although ecological factors cannot be entirely ruled out without additional data, the cultural drift hypothesis has the advantage of explaining sexual dimorphism as well. Future studies can explore the implications of these initial findings, including the extent of sex-biased dispersal between the two isolated populations (Pavan and Marroig 2016).

In conclusion, we have introduced a Bayesian coalescent-based approach to estimate *F*_*ST*_ and thereby test genetic drift as an evolutionary mechanism to explain phenotypic divergence across multiple traits. This approach directly calculates the extent of overlap between posterior distributions of *F*_*ST*_ and *P*_*ST*_. Coalescent-based analyses revealed isolated populations with minimal subsequent migration, leading to high *F*_*ST*_ values, while trait analyses showed acoustic divergence and sexual dimorphism in call frequency. Comparisons between *F*_*ST*_ and *P*_*ST*_ rejected genetic drift as a probable evolutionary mechanism behind acoustic divergence in *Pteronotus pusillus*, and somewhat less robustly in *P*. *portoricensis*. The significantly higher calls of the Hispaniolan population, together with lower calls of males make female choice an unlikely evolutionary mechanism, and instead leave open the possibility of female-mediated cultural drift. By integrating Bayesian coalescent and trait analyses, this study demonstrates a powerful approach to testing genetic drift as the key evolutionary process in trait differentiation.

### Data archiving

We have deposited the primary data underlying these analyses as follows:

- Sampling locations, morphological data, R scripts and Bayesian models available from Dryad: 10.5061/dryad.fg53g0j.
- DNA sequences: Genbank accessions *cytb*: KX787941-KX787953, KX787967-KX787994, *stat5a*: KY077747- KY077757*, plcb4*: KY077790-KY077814*, rag2*: KY077758-KY077789, and *atp7a*: KY077742-KY077746.

## Electronic supplementary material

Supplementary tables and figures

Figure S1

Figure S2

Figure S3

Figure S4

## References

[CR1] Armstrong KN, Coles RB (2007). Echolocation call frequency differences between geographic isolates of *Rhinonicteris aurantia* (Chiroptera: Hipposideridae): implications of nasal chamber size. J Mammal.

[CR2] Brommer JE (2011). Whither *Pst*? The approximation of *Qst* by *Pst* in evolutionary and conservation biology. J Evol Biol.

[CR3] Brommer JE, Hanski IK, Kekkonen J, Väisänen RA (2014). Size differentiation in Finnish house sparrows follows Bergmann’s rule with evidence of local adaptation. J Evol Biol.

[CR4] Campbell P, Pasch B, Pino JL, Crino OL, Phillips M, Phelps SM (2010). Geographic variation in the songs of neotropical singing mice: testing the relative importance of drift and local adaptation. Evolution.

[CR5] Chen SF, Jones G, Rossiter SJ (2009). Determinants of echolocation call frequency variation in the Formosanlesser horseshoe bat (*Rhinolophus monoceros*).. Proc R Soc B.

[CR6] Clare E, Adams A, Maya-Simoes A, Eger J, Hebert P, Fenton MB (2013). Diversification and reproductive isolation: cryptic species in the only New World high-duty cycle bat, *Pteronotus parnellii*. BMC Evol Biol.

[CR7] Corthals A, Martin A, Warsi OM, Woller-Skar M, Lancaster W, Russell A (2015). From the field to the lab: best practices for field preservation of bat specimens for molecular analyses. PLoS ONE.

[CR8] Dávalos LM (2006). The geography of diversification in the mormoopids (Chiroptera: Mormoopidae). Biol J Linn Soc.

[CR9] Dávalos LM, Velazco PM, Warsi OM, Smits P, Simmons NB (2014). Integrating incomplete fossils by isolatingconflicting signal in saturated and non-independent morphological characters.. Syst Biol.

[CR10] Davies KTJ, Cotton JA, Kirwan JD, Teeling EC, Rossiter SJ (2012). Parallel signatures of sequence evolution among hearing genes in echolocating mammals: an emerging model of genetic convergence. Heredity.

[CR11] Gabry J. (2017). bayesplot: Plotting for Bayesian models, v. 1.2.0. http://mc-stan.org/bayesplot/. Acessed 1 Aug 2018

[CR12] Gannon MR, Kurta A, Rodriguez Duran A, Willig MR (2005). Bats of Puerto Rico: an island focus and a Caribbean perspective.

[CR13] Gelman A, Hill J (2007). Data analysis using regression and multilevel/hierarchical models..

[CR14] Gelman A, Rubin DB (1992). Inference from iterative simulation using multiple sequences. Stat Sci.

[CR15] Gillam EH, McCracken GF (2007). Variability in the echolocation of *Tadarida brasiliensis*: effects of geography and local acoustic environment. Anim Behav.

[CR16] Guillén, Juste J, Ibáñez C (2000). Variation in the frequency of the echolocation calls of *Hipposideros ruber* inthe Gulf of Guinea: an exploration of the adaptive meaning of the constant frequency value in rhinolophoid CFbats.. J Evol Biol.

[CR17] Hedrick PW (2005). A standardized genetic differentiation measure.. Evolution.

[CR18] Hey J, Nielsen R (2004). Multilocus methods for estimating population sizes, migration rates and divergence time, with applications to the divergence of *Drosophila pseudoobscura* and *D. persimilis*. Genetics.

[CR19] Hey J, Nielsen R (2007). Integration within the Felsenstein equation for improved Markov chain Monte Carlo methods in population genetics. PNAS.

[CR20] Huffman RF, Henson OW (1990). The descending auditory pathway and acousticomotor systems: connections with the inferior colliculus. Brain Res Rev.

[CR21] Jones G (1996). Does echolocation constrain the evolution of body size in bats? In: Miller PJ (ed) Symposia of the Zoological Society of London 1960–1999. The Society, London, p 111–128.

[CR22] Kingston T, Lara MC, Jones G, Akbar Z, Kunz TH, Schneider CJ (2001). Acoustic divergence in two cryptic *Hipposideros* species: A role for social selection?. Proc R Soc B.

[CR23] Kingston T, Rossiter SJ (2004). Harmonic-hopping in Wallacea’s bats. Nature.

[CR24] Knörnschild M, Jung K, Nagy M, Metz M, Kalko E (2012). Bat echolocation calls facilitate social communication.. Proc R Soc B.

[CR25] Knowles LL, Carstens BC (2007). Delimiting species without monophyletic gene trees. Syst Biol.

[CR26] Kruschke JK (2013). Bayesian estimation supersedes the *t* test. J Exp Psychol Anim Behav Process.

[CR27] Lande R (1992). Neutral theory of quantitative genetic variance in an Island model with local extinction and colonization. Evolution.

[CR28] Leaché AD, Harris RB, Maliska ME, Linkem CW (2013). Comparative species divergence across eight triplets ofspiny lizards (*Sceloporus*) using genomic sequence data. Genome Biol Evol.

[CR29] Leinonen T, Cano JM, Mäkinen H, Merilä J (2006). Contrasting patterns of body shape and neutral genetic divergence in marine and lake populations of threespine sticklebacks. J Evol Biol.

[CR30] López-Baucells A, Torrent L, Rocha R, Pavan AC, Bobrowiec PED, Meyer CFJ (2017). Geographical variation in the high-duty cycle echolocation of the cryptic common mustached bat *Pteronotus* cf. *rubiginosus* (Mormoopidae). Bioacoustics 1–17.

[CR31] Maechler M (2016) sfsmisc, v. 1.1-0. https://CRAN.R-project.org/package=sfsmisc.

[CR32] McDonald JH, Kreitman M (1991). Adaptive protein evolution at the *Adh* locus in *Drosophila*. Nature.

[CR33] Muir G, Dixon CJ, Harper AL, Filatov DA (2012). Dynamics of drift, gene flow, and selection during speciation in *Silene*. Evolution.

[CR34] Muscarella RA, Murray KL, Ortt D, Russell AL, Fleming TH (2011). Exploring demographic, physical, and historical explanations for the genetic structure of two lineages of greater antillean bats. PLoS ONE.

[CR35] Núñez-Novas MS, León YM, Mateo J, Dávalos LM (2016). Records of the cave-dwelling bats (Mammalia: Chiroptera) of Hispaniola with an examination of seasonal variation in diversity. Acta Chiropt.

[CR36] O’Hara RB, Merilä J (2005). Bias and precision in Q estimates: problems and some solutions.. Genetics.

[CR37] Odendaal L, Jacobs D, Bishop J (2014). Sensory trait variation in an echolocating bat suggests roles for both selection and plasticity. BMC Evol Biol.

[CR38] Pavan AC, Bobrowiec PED, Percequillo AR (2018). Geographic variation in a South American clade of mormoopid bats, *Pteronotus* (*Phyllodia*), with description of a new species. J Mammal.

[CR39] Pavan AC, Marroig G (2016). Integrating multiple evidences in taxonomy: species diversity and phylogeny ofmustached bats (Mormoopidae: *Pteronotus*).. Mol Phylogenet Evol.

[CR40] Pavan AC, Marroig G (2017). Timing and patterns of diversification in the Neotropical bat genus Pteronotus(Mormoopidae).. Mol Phylogenet Evol.

[CR41] Plummer M (2003) JAGS: a program for analysis of Bayesian graphical models using Gibbs sampling. In: Hornik K, Leisch F, Zeileis A (eds) Proceedings of the 3rd International Workshop on Distributed Statistical Computing. Technische Universität Wien, Vienna, Austria, p 1–10

[CR42] Puechmaille SJ, Borissov IM, Zsebok S, Allegrini B, Hizem M, Kuenzel S (2014). Female mate choice can drive the evolution of high frequency Echolocation in bats: a case study with *Rhinolophus mehelyi*. PLoS ONE.

[CR43] Puechmaille SJ, Gouilh MA, Piyapan P, Yokubol M, Mie KM, Bates PJ (2011). The evolution of sensorydivergence in the context of limited gene flow in the bumblebee bat.. Nat Commun.

[CR44] Rojas D, Warsi OM, Dávalos LM (2016). Bats (Chiroptera: Noctilionoidea) challenge a recent origin of extant neotropical diversity. Syst Biol.

[CR45] Russell AL, Goodman SM, Cox MP (2008). Coalescent analyses support multiple mainland-to-island dispersals in the evolution of Malagasy *Triaenops* bats (Chiroptera: Hipposideridae). J Biogeogr.

[CR46] Schipper J, Chanson JS, Chiozza F, Cox NA, Hoffmann M, Katariya V (2008). The status of the world’s land and marine mammals: diversity, threat, and knowledge. Science.

[CR47] Spitze K (1993). Population structure in *Daphnia obtusa*: quantitative genetic and allozymic variation. Genetics.

[CR48] Su Y-S, Yajima M. (2012). R2jags: A Package for Running jags from R, v. 0.5-7. https://cran.r-project.org/web/packages/R2jags/index.html.

[CR49] Sundqvist L, Keenan K, Zackrisson M, Prodöhl P, Kleinhans D (2016). Directional genetic differentiation and relative migration. Ecol Evol.

[CR50] Takahata N (1983). Gene identity and genetic differentiation of populations in the Finite Island Model.. Genetics.

[CR51] Whitlock MC, McCauley DE (1999). Indirect measures of gene flow and migration: F ≠1/(4Nm+1).. Heredity.

[CR52] Winter DJ (2012). mmod: an R library for the calculation of population differentiation statistics. Mol Ecol Resour.

[CR53] Woerner AE, Cox MP, Hammer MF (2007). Recombination-filtered genomic datasets by information maximization. Bioinformatics.

[CR54] Wright S (1931). Evolution in Mendelian populations. Genetics.

[CR55] Wright S (1951). The genetical structure of populations. Ann Eugen.

[CR56] Wright S (1965). The interpretation of population structure by F‐statistics with special regard to systems of mating. Evolution.

[CR57] Yoshino H, Armstrong KN, Izawa M, Yokoyama JUN, Kawata M (2008). Genetic and acoustic population structuring in the Okinawa least horseshoe bat: are intercolony acoustic differences maintained by vertical maternal transmission?. Mol Ecol.

