# Supplementary material for: A coalescent-based estimator of genetic drift, and acoustic divergence in the Pteronotus parnellii species complex
Source: Heredity (Edinb). 2018 Aug 17;122(4):417–27. doi: 10.1038/s41437-018-0129-3 (PMC6460761; doi:10.1038/s41437-018-0129-3)
Supplement: Supplementary file 1 — Supplementary tables and figures [file 41437_2018_129_MOESM1_ESM.docx]

**Supplementary Table 1**. Location, sex, echolocation, and measurements of individuals sampled. Body measurements, except for body mass, are in mm.

| Individual | Cave | Island | Median frequency (Hz) | Sex | Length | Tail | Ear | Foot | Forearm | Body mass (g) |
| --- | --- | --- | --- | --- | --- | --- | --- | --- | --- | --- |
| ALR108 | del Agua | Hispaniola | 67214 | M | 55 | 16 | 11 | 9 | 51 | 9.4 |
| ALR109 | del Agua | Hispaniola | 66944 | M | 51 | 17 | 16 | 8 | 49 | 10.6 |
| ALR110 | del Agua | Hispaniola | 68010 | F | 53 | 21 | 13 | 13 | 50 | 11.6 |
| ALR111 | del Agua | Hispaniola | 68121 | F | 54 | 14 | 12 | 7 | 51 | 10.1 |
| ALR112 | del Agua | Hispaniola | 68547 | F | 54 | 14 | 14 | 6 | 48 | 11.5 |
| ALR113 | del Agua | Hispaniola | 68340 | F | 57 | 15 | 21 | 6 | 52 |  |
| ALR114 | del Agua | Hispaniola | 66877 | M | 53 | 13 | 19 | 9 | 51 | 8.8 |
| ALR115 | del Agua | Hispaniola | 68359 | M | 55 | 18 | 21 | 9 | 49 | 8.6 |
| ALR116 | del Agua | Hispaniola | 68010 | F | 54 | 16 | 21 | 10 | 50 | 11.6 |
| ALR117 | del Agua | Hispaniola | 67436 | F | 54 | 19 | 18 | 10 | 51 | 11.5 |
| ALR118 | del Agua | Hispaniola | 68467 | M | 53 | 18 | 18 | 9 | 50 |  |
| ALR122 | del Agua | Hispaniola | 68422 | F | 53 | 18 | 17 | 9 | 51 | 13.4 |
| ALR137 | Honda de Julian | Hispaniola | 67879 | F | 58 | 27 | 15 | 12 | 49 | 11.5 |
| ALR138 | Honda de Julian | Hispaniola | 67604 | F | 59 | 21 | 13 | 11 | 51 | 8.9 |
| ALR139 | Honda de Julian | Hispaniola | 67728 | F | 54 | 18 | 18 | 8 | 51 | 13.1 |
| ALR140 | Honda de Julian | Hispaniola | 66800 | F | 51 | 22 | 21 | 9 | 49 | 11.9 |
| ALR142 | Honda de Julian | Hispaniola | 66688 | F | 52 | 18 | 9 | 8 | 51 | 12.3 |
| ALR143 | Honda de Julian | Hispaniola | 68250 | F | 50 | 18 | 11 | 10 | 51 | 10.8 |
| ALR147 | Honda de Julian | Hispaniola | 66942 | M | 54 | 26 | 14 | 11 | 50 |  |
| ALR148 | Honda de Julian | Hispaniola | 68905 | F | 57 | 18 | 12 | 9 | 48 | 12.3 |
| ALR149 | Honda de Julian | Hispaniola | 69834 | F | 54 | 17 | 12 | 10 | 52 | 8.5 |
| ALR150 | Honda de Julian | Hispaniola | 68065 | F | 54 | 11 | 14 | 8 | 48 | 11.6 |
| ALR152 | Honda de Julian | Hispaniola |  | M | 54 | 16 | 12 | 9 | 49 | 9.9 |
| ALR153 | Honda de Julian | Hispaniola | 69124 | F | 56 | 16 | 12 | 11 | 47 | 7.5 |
| ALR154 | Honda de Julian | Hispaniola | 68751 | F | 56 | 14 | 13 | 9 | 50 | 12.1 |
| ALR162 | La Chepa | Hispaniola | 67303 | M | 47 | 22 | 14 | 11 | 51 | 11.7 |
| ALR163 | La Chepa | Hispaniola | 67303 | M | 55 | 25 | 13 | 9 | 51 | 10.1 |
| ALR164 | La Chepa | Hispaniola | 67431 | M | 56 | 16 | 12 | 11 | 52 |  |
| ALR165 | La Chepa | Hispaniola | 67736 | M | 56 | 20 | 16 | 9 | 50 | 9.9 |
| ALR166 | La Chepa | Hispaniola | 66926 | M | 52 | 23 | 14 | 11 | 52 | 10.8 |
| ALR167 | La Chepa | Hispaniola | 66844 | M | 54 | 21 | 17 | 11 | 51 | 10.1 |
| ALR168 | La Chepa | Hispaniola | 67992 | M | 54 | 18 | 14 | 11 | 50 | 10.1 |
| ALR169 | La Chepa | Hispaniola | 66441 | M | 54 | 18 | 11 | 11 | 52 | 10.3 |
| ALR171 | La Chepa | Hispaniola | 66229 | M | 54 | 22 | 18 | 14 | 51 | 9.8 |
| ALR172 | La Chepa | Hispaniola | 68272 | M | 50 | 18 | 14 | 12 | 48 | 10.1 |
| ALR188 | La Chepa | Hispaniola | 67455 | F | 55 | 21 | 13 | 11 | 49 |  |
| ALR189 | La Chepa | Hispaniola |  | F | 55 | 24 | 13 | 11 | 51 | 12.0 |
| ALR190 | La Chepa | Hispaniola | 68010 | F | 58 | 16 | 14 | 10 | 49 | 10.7 |
| ALR021 | Bonita | Puerto Rico | 62212 | F | 63 | 18 | 16 | 11 | 55 | 14.0 |
| ALR025 | Bonita | Puerto Rico | 62116 | F | 55 | 15 | 14 | 7 | 53 | 12.5 |
| ALR027 | Bonita | Puerto Rico | 62125 | F | 56 | 15 | 14 | 11 | 50 | 12.0 |
| ALR029 | Bonita | Puerto Rico | 62546 | F | 55 | 8 | 12 | 12 | 55 |  |
| ALR031 | Bonita | Puerto Rico | 62324 | M | 60 | 12 | 13 | 8 | 51 | 12.5 |
| ALR032 | Bonita | Puerto Rico | 62190 | M | 52 | 17 | 16 | 9 | 49 | 12.5 |
| ALR033 | Bonita | Puerto Rico | 63058 | M | 54 | 19 | 19 | 9 | 49 | 12.5 |
| ALR035 | Bonita | Puerto Rico | 61937 | M | 58 | 18 | 14 | 5 | 50 | 13.0 |
| ALR036 | Bonita | Puerto Rico | 62859 | F | 55 | 21 | 13 | 10 | 52 | 14.5 |
| ALR037 | Bonita | Puerto Rico | 62496 | F | 60 | 14 | 14 | 11 | 54 | 14.5 |
| ALR038 | Bonita | Puerto Rico | 61932 | M | 62 | 17 | 19 | 9 | 51 | 15.5 |
| ALR039 | Bonita | Puerto Rico | 62886 | M | 52 | 19 | 18 | 11 | 51 | 15.0 |
| ALR040 | Bonita | Puerto Rico | 62950 | M | 55 | 18 | 12 | 13 | 52 | 15.0 |
| ALR041 | Culebrones | Puerto Rico | 63144 | F |  |  |  |  |  | 14.0 |
| ALR042 | Culebrones | Puerto Rico | 61854 | M |  |  |  |  |  | 13.0 |
| ALR043 | Culebrones | Puerto Rico | 62212 | F |  |  |  |  |  | 12.5 |
| ALR075 | Culebrones | Puerto Rico | 60451 | M | 55 | 22 | 11 | 12 | 52 | 13.0 |
| WCL001 | Culebrones | Puerto Rico | 62296 | M | 55.4 | 17 | 9.9 | 12.8 | 52.2 | 11.1 |
| WCL002 | Culebrones | Puerto Rico | 62154 | F | 60.6 | 14.7 | 9.6 | 12.7 | 51.7 | 12.4 |
| ALR087 | Volcan | Puerto Rico | 61035 | F | 52 | 17 | 17 | 11 | 52 | 11.5 |
| WCL003 | Volcán | Puerto Rico | 61323 |  | 63.1 | NA | 10.3 | 11.3 | 52.5 | 12.0 |
| WCL004 | Volcán | Puerto Rico | 61726 |  | 57.2 | NA | 8.4 | 11 | 49.8 | 11.1 |
| WCL005 | Volcán | Puerto Rico | 61935 | M | 57.4 | 19 | 11 | 11.3 | 53.9 | 12.5 |
| Mean (s.d.) |  | Hispaniola | 67330.0 (672.0) | M | 53.4 (2.3) | 19.2 (3.5) | 14.9 (2.9) | 10.2 (1.5) | 50.4 (1.2) | 10.0 (0.8) |
| Mean (s.d.) |  | Hispaniola | 68099.0 (743.8) | F | 54.7 (2.3) | 18.0 (3.7) | 14.6 (3.4) | 9.4 (1.8) | 50.0 (1.4) | 11.2 (1.5) |
| Mean (s.d.) |  | Puerto Rico | 62164.8 (719) | M | 56.1 (3.3) | 17.8 (2.5) | 14.3 (3.5) | 10.0 (2.5) | 51.1 (1.5) | 13.2 (1.4) |
| Mean (s.d.) |  | Puerto Rico | 62289.9 (560.2) | F | 57.1 (3.7) | 15.3 (3.7) | 13.7 (2.3) | 10.7 (1.7) | 52.8 (1.8) | 13.1 (1.1) |

**Supplementary Table 2**. Geographical locations for sampling sites.

| Cave | Island | Latitude | Longitude |
| --- | --- | --- | --- |
| del Agua | Hispaniola | 18.408 | -68.882 |
| Honda de Julian | Hispaniola | 19.132 | -70.079 |
| La Chepa | Hispaniola | 18.869 | -69.576 |
| Bonita | Puerto Rico | 18.375 | -66.305 |
| Culebrones | Puerto Rico | 18.418 | -66.729 |
| Volcán | Puerto Rico | 18.332 | -66.524 |

**Supplementary Table 3.** Diversity statistics and tests of selection for all genetic loci. McDonald-Kreitman (MK) tests were run using *P. rubiginosus* as an outgroup. MK tests of selection were not applicable to *plcb4* and *stat5a* as they are noncoding loci (5' UTR and intron regions, respectively).

| Locus | Sample size | Size (bp) | Segregating sites | McDonald-Kreitman test results |
| --- | --- | --- | --- | --- |
| *atp7a* | 10 | 637 | 1 | not significant; *P* = 0.362 |
| *plcb4* | 50 | 307 | 9 | NA |
| *rag2* | 62 | 472 | 2 | not significant; *P* = 0.082 |
| *stat5a* | 22 | 429 | 3 | NA |
| *cytb* | 32 | 1121 | 64 | not significant; *P* = 0.350 |

**Supplementary Table 4**. Posterior densities for IMa2 analyses of *Pteronotus pusillus* and *P. portoricensis* species pair. Divergence times are given in thousands of years, and unidirectional migration rates are given per generation.

| Parameter | Point estimate | 95% confidence interval |
| --- | --- | --- |
| θ_DR_ | 0.625 | (0.225, 1.975) |
| *N*_e_ (Hispaniola) | 137,679 | (49564, 435066) |
| θ_PR_ | 0.125 | (0.025, 0.575) |
| *N*_e_ (Puerto Rico) | 27,536 | (5507, 126665) |
| τ | 0.703 | (0.258, 4.793) |
| Divergence time (kya) | 1,238 | (454, 8447) |
| *m*_DR_ (PR → DR) | 1.875 | (0.475, 15.07) |
| Nm_DR_ (PR → DR) | 0.325 | (0.077, 2.336) |
| *m*_PR_ (DR → PR) | 0.025 | (0.025, 30.52) |
| Nm_PR_ (DR → PR) | 0.049 | (0.007, 0.907) |

**Supplementary Table 5**. Posterior estimates of call frequency *P_ST_* with sample-wide effects of sex. The sex effect is coded with females as the baseline, the effect shown is for males. *c*/*h^2^*, critical value for the *P_ST_* vs. *F_ST_* comparison of the proportion of heritability ascribable to the additive genetic variance; His., Hispaniola; HPD, high probability density; P.R., Puerto Rico.

| Variable | His. *c*/*h^2^* | P.R. *c*/*h^2^* | Mean | 2.5% HPD | Median | 97.5% HPD |
| --- | --- | --- | --- | --- | --- | --- |
| Sample-wide sex *P_ST_* | 0.358 | >1 | 0.984 | 0.879 | 0.999 | 1.000 |
| Sample-wide sex effect | – | – | -0.521 | -0.907 | -0.515 | -0.136 |

**Supplementary Figure 1**. Results from IMa2 analyses of *Pteronotus parnellii* s.l. populations. Joint posterior density of the effective number of migrants per generation from one population to the other.

**Supplementary Figure 2**. Call frequency as a function of body dimensions. Analyses of covariance support very different call frequency intercepts for island groups (*F_(1, 44)_* = 545.532, *P*-value = 0.000), little influence of principal component (PC) 1 (*F_(1, 44)_* = 0.258, *P*-value = 0.614), and the influence of PC 2 (PC2, *F_(1, 44)_* = 7.390, *P*-value = 0.009) on echolocation call frequency. Bayesian 95% high-probability density (HDP) of the difference in PC1 between Puerto Rico and Hispaniola was -2.63, -0.98, and 0.11-1.10 for PC2.

**Supplementary Figure 3**. Posterior predictive simulations of the standard deviation including the effect of sex as an island-specific (left, see Table 1), or sample-wide (right) factor.

**Supplementary Figure 4**. Densities of Bayesian posteriors for *F_ST_* based on between-population migration rates, and *P_ST_* for call frequency with sample-wide (as opposed to island-specific) sex effects. The lines show the 95^th^ percentile for the corresponding *F_ST_*, and the 5% percentile for the *P_ST_*.. The overlap between *P_ST_* call frequency and *F_ST_* Hispaniola was 0.0006, for *F_ST_* Puerto Rico it was 0.006.
