# Supplementary figures and images for: A coalescent-based estimator of genetic drift, and acoustic divergence in the Pteronotus parnellii species complex
Source: Heredity (Edinb). 2018 Aug 17;122(4):417–27. doi: 10.1038/s41437-018-0129-3 (PMC6460761; doi:10.1038/s41437-018-0129-3)

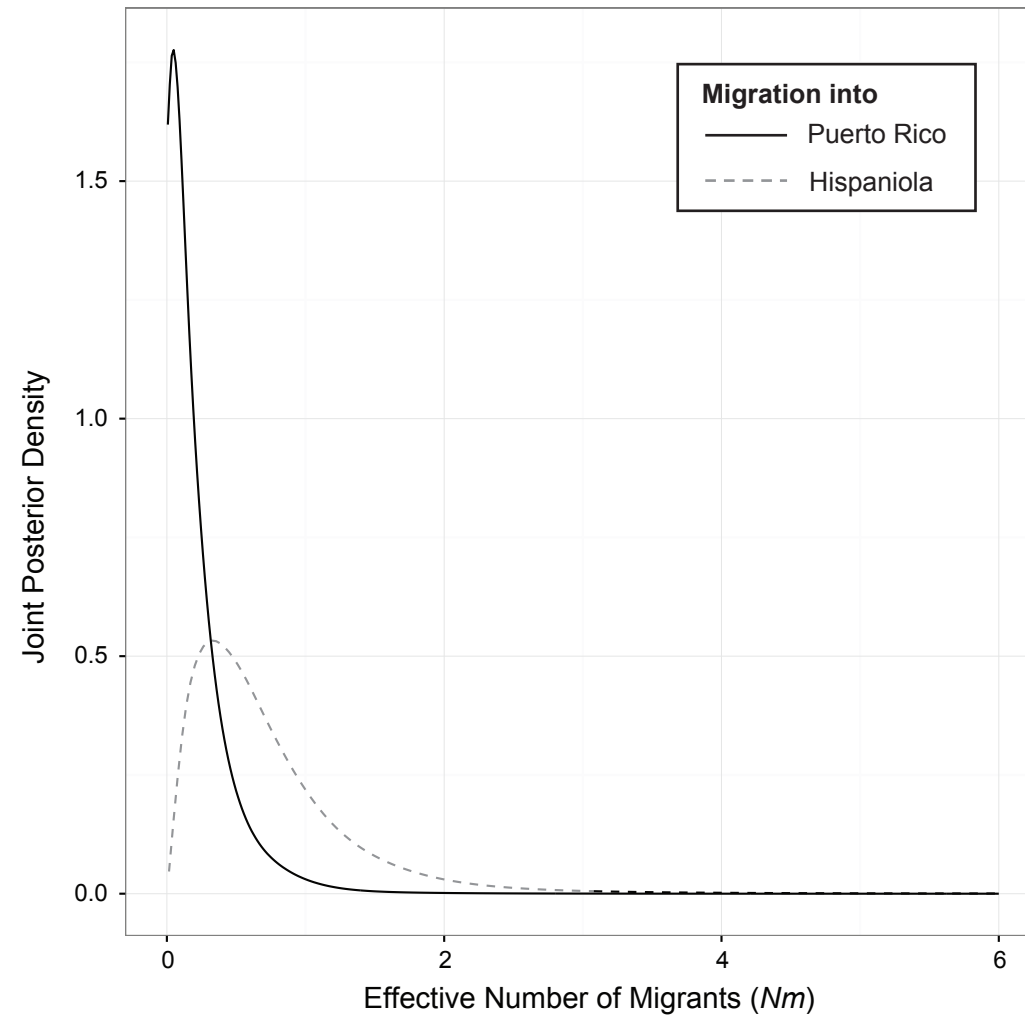

Supplementary Figure 1

Supplement: Supplementary file 2 — Figure S1 [file 41437_2018_129_MOESM2_ESM.pdf]

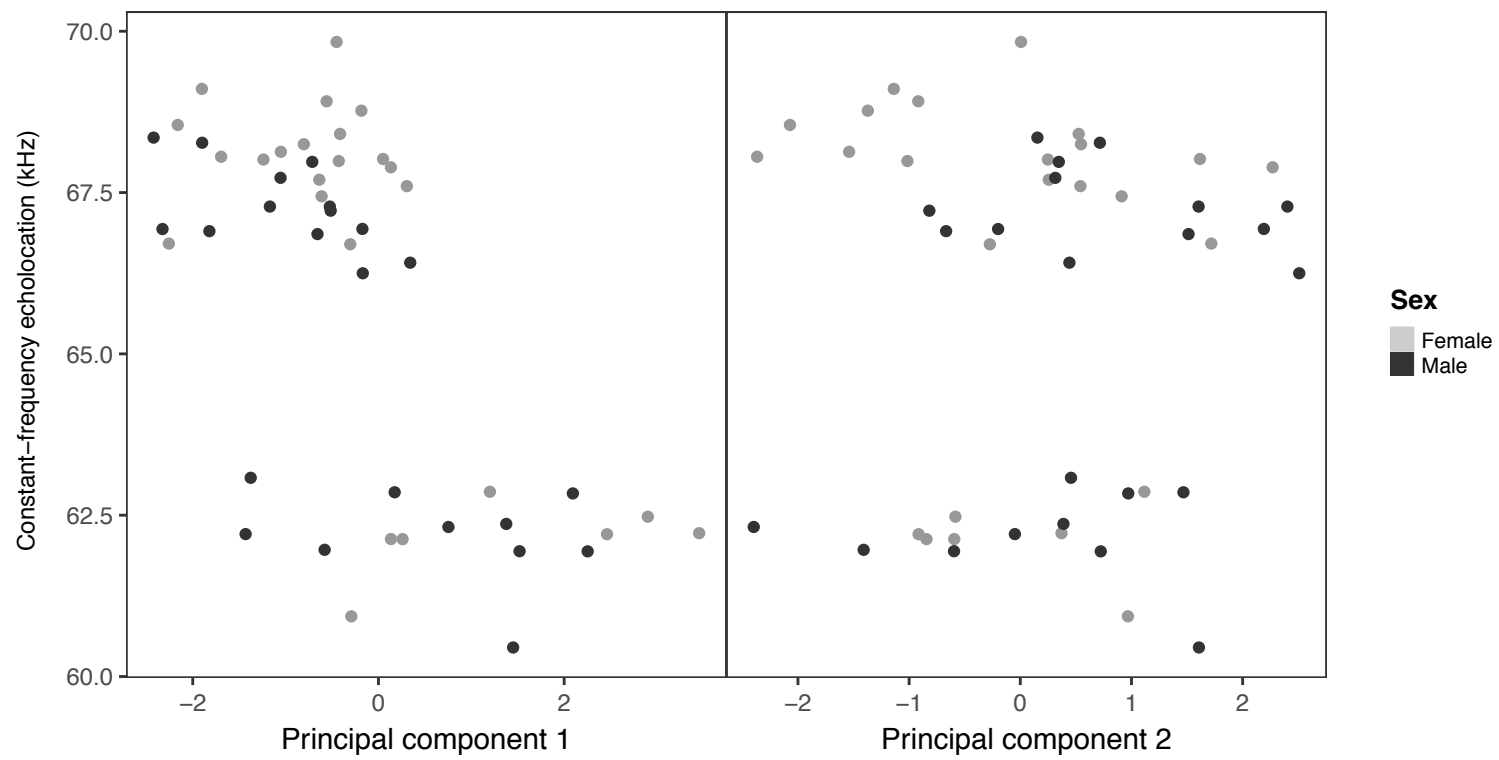

Supplementary Figure 2

Supplement: Supplementary file 3 — Figure S2 [file 41437_2018_129_MOESM3_ESM.pdf]

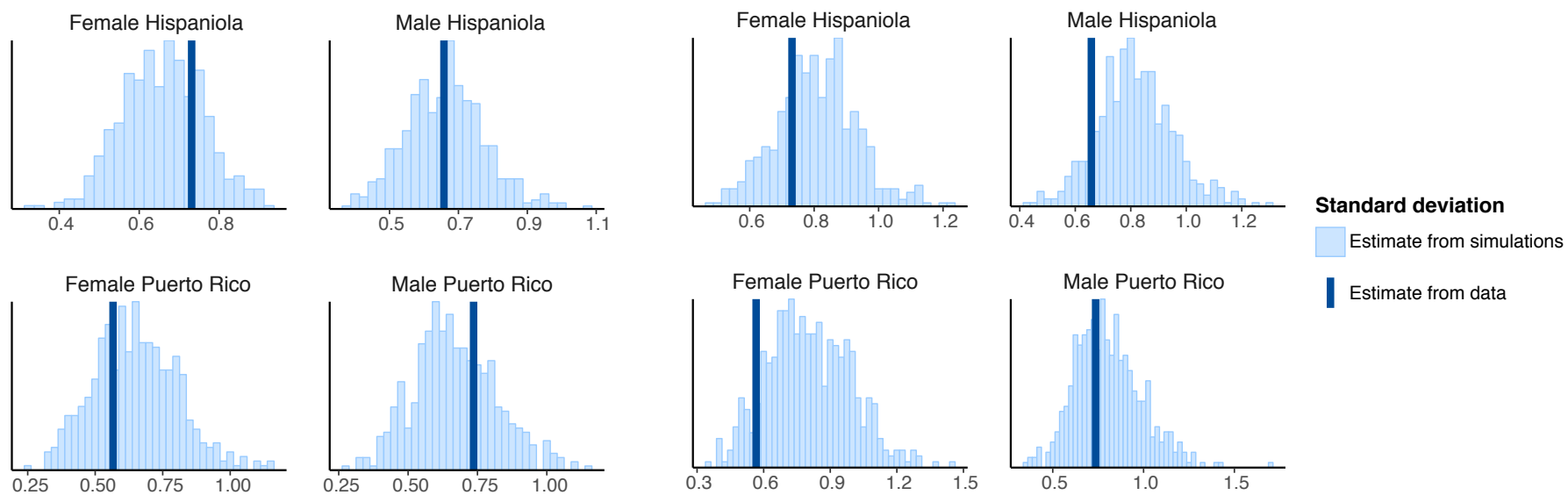

Supplementary Figure 3

Supplement: Supplementary file 4 — Figure S3 [file 41437_2018_129_MOESM4_ESM.pdf]

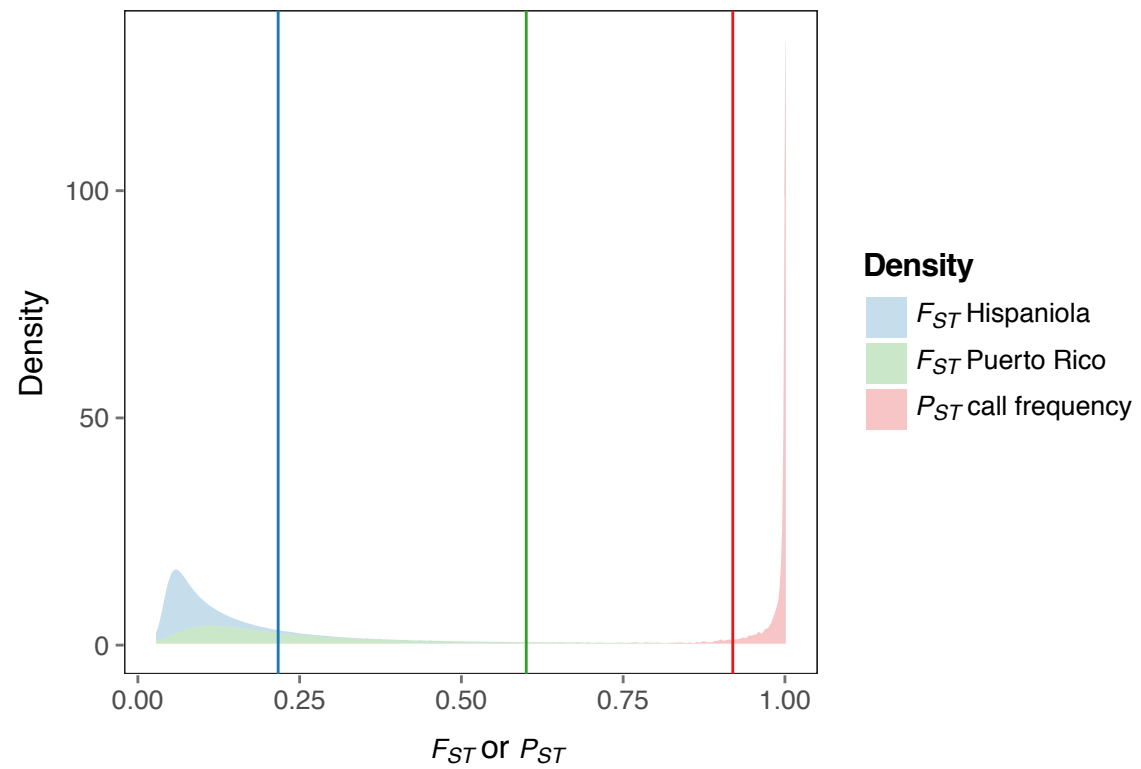

Supplementary Figure 4

Supplement: Supplementary file 5 — Figure S4 [file 41437_2018_129_MOESM5_ESM.pdf]
